# Vision In Stroke cohort: Profile overview of visual impairment

**DOI:** 10.1002/brb3.771

**Published:** 2017-10-06

**Authors:** Fiona J. Rowe, the VIS writing Group, David Wright, Darren Brand, Tallat Maan, Sarah Peel, Nicola Akerman, Caroline Dodridge, Claire Howard, Tracey Shipman, Una Sperring, Sonia MacDiarmid, Cicely Freeman

**Affiliations:** ^1^ University of Liverpool Liverpool UK

**Keywords:** central vision, ocular motility, stroke, visual field loss, visual impairment, visual inattention, visual perception, visual symptoms

## Abstract

**Aim:**

To profile the full range of visual disorders from a large prospective observation study of stroke survivors referred by stroke multidisciplinary teams to orthoptic services with suspected visual problems.

**Methods:**

Multicenter prospective study undertaken in 20 acute Trust hospitals. Standardized screening/referral forms and investigation forms documented data on referral signs and symptoms plus type and extent of visual impairment.

**Results:**

Of 1,345 patients referred with suspected visual impairment, 915 were recruited (59% men; mean age at stroke onset 69 years [SD 14]). Initial visual assessment was at median 22 days post stroke onset. Eight percent had normal visual assessment. Of 92% with confirmed visual impairment, 24% had reduced central visual acuity <0.3 logMAR and 13.5% <0.5 logMAR. Acquired strabismus was noted in 16% and acquired ocular motility disorders in 68%. Peripheral visual field loss was present in 52%, most commonly homonymous hemianopia. Fifteen percent had visual inattention and 4.6% had other visual perceptual disorders. Overall 84% were visually symptomatic with visual field loss the most common complaint followed by blurred vision, reading difficulty, and diplopia. Treatment options were provided to all with confirmed visual impairment. Targeted advice was most commonly provided along with refraction, prisms, and occlusion.

**Conclusions:**

There are a wide range of visual disorders that occur following stroke and, frequently, with visual symptoms. There are equally a wide variety of treatment options available for these individuals. All stroke survivors require screening for visual impairment and warrant referral for specialist assessment and targeted treatment specific to the type of visual impairment.

## 1 INTRODUCTION

Poststroke visual impairment occurs frequently with an estimated prevalence of 65% in an acute stroke population (Hepworth et al., 2015). Visual impairment in this population can be broadly categorized into four impaired visual functions of: reduced central vision, peripheral visual field loss, eye movement disorders, and visual perceptual disorders (Jones & Shinton, 2006). Each category also comprises a range of visual deficits specific to that visual function.

Many visual impairments cause visual symptoms with stroke survivors aware of blurred/altered vision or jumbled/double images. These visual symptoms cause impact to general function and to daily life (Hepworth & Rowe, 2016). However, a wide variety of interventions are available to aid and/or ameliorate these symptoms (Pollock et al., 2011a, 2011b, 2012).

The purpose of this study was to profile the full range of visual disorders from a large, prospective, observation cohort study of stroke survivors with suspected visual impairment, referred by stroke multidisciplinary teams to orthoptic services.

## 2 METHODS

### 2.1 Study design and population

This prospective multicenter observational case cohort study comprised local orthoptic principal investigators from 20 UK hospital trusts responsible for assessing stroke patients and collecting patient data. Orthoptic services for stroke units in the UK provide comprehensive visual assessment at the bedside and typically initiate management options at this acute stage. Review visits continue on the stroke unit as needed and, subsequently, in out‐patient eye clinics. The data were collated centrally at the University of Liverpool. The study had multicenter ethical approval via the National Research Ethics Service (06/Q0904/5) and was undertaken in accordance with the Tenets of Helsinki.

The target population was stroke patients suspected of having a visual difficulty. Referrals could be made from in‐patient wards, rehabilitation units, community services, or out‐patient clinics. Patients were given an information sheet and recruited after informed, written consent. Patients were excluded if they were unable to consent due to cognitive impairment, unwilling to consent, if their diagnosis was that of transient ischemic attack or if they were discharged without vision assessment.

### 2.2 Measures

**Figure 1:**
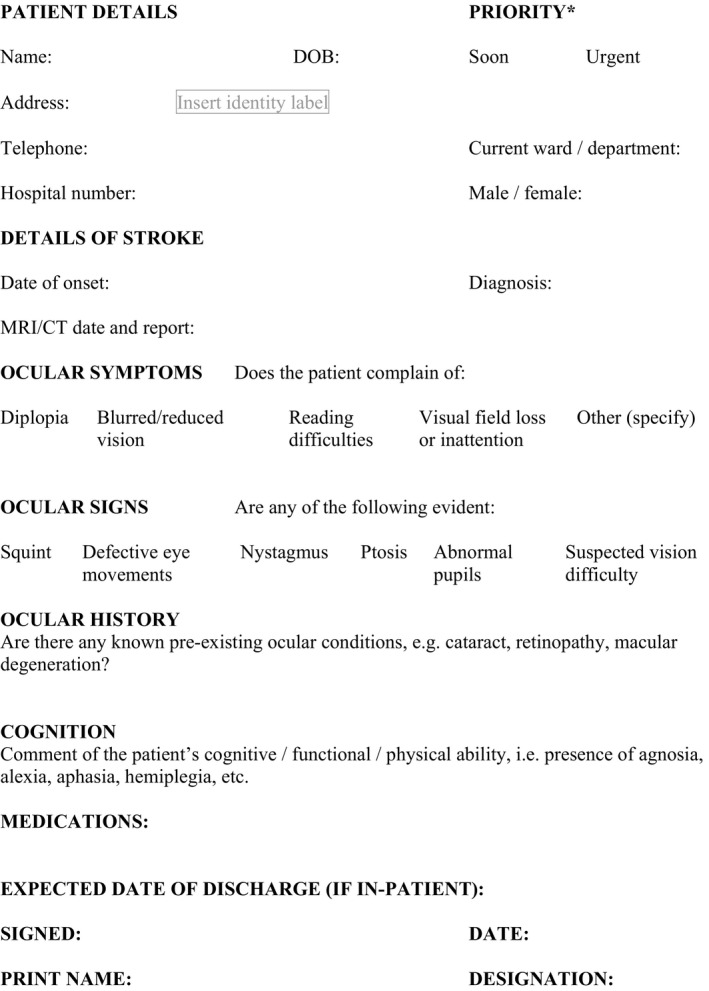
Screening/Referral form for Orthoptic examination

Patients with suspected visual difficulty were identified using a screening form (Figure 1: SPSS: RRID: SCR_002865). Subsequently this was used as the referral form to the Orthoptic service. A standardized investigation sheet was used for the eye assessment consisting of identification of known preexistent ocular pathology, symptoms and signs, investigation of visual field, ocular motility, and perceptual aspects (Rowe, 2011). Visual fields were assessed qualitatively by traditional confrontation methods or quantitatively by Humphrey (Humphrey systems, Dublin, CA, USA) automated central and/or peripheral static perimetry or Goldmann/Octopus (Haag Streit Int, Switzerland) kinetic perimetry.

Visual acuity was assessed uniocularly at near and distance fixation with Snellen or logMAR acuity tests. Low visual acuity was considered in two categories. The first defined low visual acuity as less than best corrected 6/12 Snellens acuity or 0.3 logMAR in accordance with UK driving standards. The second defined low visual acuity as less than 6/18 Snellens acuity or 0.5 logMAR and equal or better than 3/60 Snellens acuity as per World Health Organisation (WHO) guidelines.

Assessment of ocular alignment and motility consisted of cover test, evaluation of saccadic, smooth pursuit and vergence eye movements, retinal correspondence (Bagolini glasses), fusional vergence (20D or fusional range), stereopsis (Frisby near test), prism cover test, and lid and pupil function.

Perceptual deficits were recorded after questioning of the patient and/or carers and relatives. Inattention was assessed by means of a combination of assessments including line bisection, Albert's test, cancellation tests, and memory tests using verbal description and drawing. Alexia was diagnosed where patients described an inability to read (despite being able to see the text) because of being unable to decipher the words or their meaning or being unable to make sense of the text.

Quality of life was undertaken using the Activities of Daily Living Dependent on Vision (ADLDV) questionnaire. This consists of 22 questions related to vision including visual recognition, personal care and hygiene, mobility, and reading. It uses a Likert scale of 1–4 indicating the individual cannot see to do through to having no difficulty. A full “normal” score is 88.

Stroke details were recorded from patient notes accounting for stroke laterality, type, and area involved. Ocular treatment details were recorded along with outcome. Reasons for nonattendance at review appointments included death, a move out of area, lost to follow‐up, follow‐up unwanted, or unknown.

### 2.3 Data analysis

Results were inputted to the statistical package SPSS version 22 (IBM SPSS Statistics, USA). Pearson chi squared test (*x* ^2^) was undertaken to analyze cross tabulations of results for visual field loss and outcome of follow‐up versus factors such as age, presence of other visual impairment, laterality, and area of stroke and recovery. A *t* test was used to analyze differences between similar measurements with normal distributions, for example, strabismus.

## 3 RESULTS

### 3.1 General demographics

One thousand three hundred and forty‐five patients were referred for visual assessment for this study. All were suspected of having visual problems. Nine hundred and fifteen patients were recruited and 430 patients were excluded. Reasons for exclusion included inability/unwilling to provide informed, written consent as required of the ethical approval for this study (*n* = 259), patients were discharged prior to receiving visual assessment (*n* = 52), diagnosis was changed to transient ischemic attack or other pathology (*n* = 54), patients died prior to visual assessment (*n* = 26), or patients failed to attend for visual assessment (*n* = 4). It was not possible to obtain full visual information on these excluded patients.

Of 915 patients recruited, 59% (*n* = 540) were men and 41% (*n* = 375) women. Mean age at onset of stroke was 69 years (range 1–94: SD 14 years). One patient was aged 1 year and the range thereafter was 19–94 years. The median age at onset of stroke was 71 years.

Median duration from onset of stroke to initial baseline eye examination was 22 days (0–2543 days), the mean of 40.84 (SD 141.28) days being skewed by three outliers who were referred a number of years after the stroke onset. Stroke lesion was right sided in 448 patients (49%, i.e., right sided brain), left sided in 348 (38%), and bilateral in 119 (13%). Infarcts accounted for 773 cases (84.5%) with the remainder due to hemorrhage.

Overall, 8% (*n* = 72) had normal visual assessment. Of those formally diagnosed with visual impairment, 45.5% (*n* = 415) had solely one form of visual impairment: 17% (*n* = 155) had visual field loss, 20% (*n* = 181) had eye movement abnormalities, 2% (*n* = 17) had visual perceptual difficulties, and 7% (*n* = 62) had low central vision—whereas 46.5% (*n* = 423) had multiple visual impairments.

### 3.2 Central vision

Median visual acuity for each eye was 0.2 logMAR with a mean of 0.26 (SD 0.39, range −0.2–2.5). About 32% (*n* = 296) had visual acuity of 0.0 logMAR (6/6 Snellen equivalent) or better, 76% had visual acuity of 0.3 logMAR (6/12 Snellen) or better (*n* = 698), and 86.5% had visual acuity of 0.5 logMAR (6/18 Snellen) or better (*n* = 792). Three quarters of the cohort required glasses (75.6%, *n* = 692), 5% had preexistent strabismus and/or amblyopia and 27.4% had coexistent ocular pathology; typically glaucoma, age‐related macular degeneration, cataract, and diabetic retinopathy (Table 1).

**Table 1:** Types of coexistent ocular pathology

Number of patients	Cataract	Retinopathy	Age‐related macular degeneration	Glaucoma	Pupil anomaly	Color defect	Artificial eye	Corneal anomaly
Patients with sole ocular pathology	26	6	2	19	0	0	0	0
Patients with multiple ocular pathologies	102	38	20	22	3	2	2	2

A further seven patients were registered partially sighted.

### 3.3 Ocular alignment and movement

Manifest strabismus was noted in 18.5% (*n* = 169) patients of which 3% was long‐standing prior to the stroke onset (Table 2A). Acquired constant exotropia occurred most frequently, *p* = .001.

**Table 2:** Eye movement disorders

**A Strabismus types**						
Exotropia	Esotropia	Hypertropia	Hypotropia	Eso and hypotropia	Exo and hypotropia	Skew deviation
68	44	16	12	1	22	6
**B Ocular motility disorders**						
		III nerve palsy	IV nerve palsy	VI nerve palsy	Ophthalmoplegia	Impaired gaze holding
Patients with sole motility disorder		9	8	24	3	37
Patients with multiple motility disorders		11	4	28	0	9
		Complete gaze palsy	Horizontal gaze palsy	Vertical gaze palsy	Dorsal midbrain syndrome	INO/one and a half syndrome
Patients with sole motility disorder		15	0	0	5	9
Patients with multiple motility disorders		8	16	17	3	11
		Saccadic palsy	Saccadic dysmetria	Smooth pursuit palsy	Impaired depression	Impaired elevation
Patients with sole motility disorder		17	206	18	3	0
Patients with multiple motility disorders		11	72	28	3	42
**C Nystagmus types**						
Upbeat		Pendular	Horizontal	Downbeat	Rotary	Multivector
11		1	12	7	6	7
Pathological end‐point		Abducting	Retraction	Gaze evoked	Latent	Idiopathic
31		9	8	17	1	2

Numbers of patients.

Ocular motility abnormalities were documented in 68% (*n* = 622) patients (Table 2B); most frequently saccadic dysmetria (30.4%, *n* = 278), gaze defects (22.6%, *n* = 207), and cranial nerve palsies (9.7%, *n* = 89). A variety of nystagmus types were recorded in 12.2% (*n* = 112) with the most common type being pathological end‐point nystagmus (Table 2C).

Normal convergence near point of 6 cm was reported in nearly 39% of patients (*n* = 354). Reduced near point of convergence less than 8 cm was recorded in nearly 38% (*n* = 345) and for less than 10 cm in 26.7% (*n* = 235).

### 3.4 Lid and pupil function

Normal lid function was evident in 85.9% (*n* = 786). The remainder had unilateral or bilateral ptosis or lid retraction, with unilateral ptosis being most common (Table 3A). Normal pupil function was evident in 90.8% (*n* = 831) with the remainder having varied forms of dilated or miosed pupils (Table 3B). Relative afferent pupillary defect and anisocoria were the most common forms.

**Table 3:** Lid and pupil disorders

**A Lid function disorders**				
Unilateral ptosis	Bilateral ptosis	Unilateral lid retraction	Bilateral lid retraction	Senile ptosis
80	8	5	5	5
**B Pupil disorders**				
Relative afferent pupillary defect	Light‐near dissociation	Anisocoria	Middilated pupils	Miosed pupils
14	3	15	6	8
Sluggish pupils	Horner's syndrome	Adie's pupil	Coloboma	
4	5	1	1	

Numbers of patients.

### 3.5 Visual field loss and visual perception

Over half (52.3%, *n* = 479) of patients had visual field loss (Table 4). The most common type of visual field loss was found to be complete (*n* = 259) and partial (*n* = 79) homonymous hemianopia and occurring significantly more frequently to the left side than to the right side or bilaterally, *p* = .001 (*t* test). Other types included superior or inferior quadrantanopia (*n* = 73), constricted visual fields (*n* = 44), scotomas (*n* = 5), temporal crescent defect (*n* = 1), and bilateral hemianopia (cortical blindness: *n* = 1).

**Table 4:** Types of visual field loss

Complete homonymous hemianopia	Partial homonymous hemianopia	Macular sparing homonymous hemianopia	Superior quadrantanopia	Inferior quadrantanopia	Chequerboard quadrantanopia	Constricted visual fields
259	79	5	30	40	3	44
Scotoma	Altitudinal	Bilateral homonymous hemianopia	Spared temporal crescent	Homonymous hemianopia and contralateral quadrantanopia	Binasal hemianopia	Unilateral blind eye
5	3	1	1	6	1	2

Numbers of patients.

Visual inattention was noted in 15% of the cohort (*n* = 137) whereas other visual perceptual deficits such as visual agnosia, cortical color visual or depth impairment, and acquired alexia were noted in 4.6%.

### 3.6 Symptoms

Visual symptoms (Table 5) were reported by 84% of patients (*n* = 766) either as a sole symptom (56%) or combined as two or more visual symptoms (28%). Visual field loss was the most common symptom (45.6%) followed by blurred vision (31.2%), reading difficulty (19.6%), and diplopia (17.3%). Visual field loss was typically reported in the presence of visual field loss, diplopia was typical for ocular alignment and/or motility disorders, whereas blurred vision and reading difficulty were nonspecific symptoms of various ocular diagnoses. Fifty patients had a normal visual examination despite having reported visual symptoms.

**Table 5:** Visual symptoms

	Visual field loss	Blurred vision	Reading difficulty	Diplopia	Visual hallucination	Oscillopsia	Perceptual difficulties
Patients reporting one primary symptom *N* = 511/766 (56%)	227 (24.8%)	134 (14.6%)	39 (4.3%)	84 (9.2%)	13 (1.4%)	1 (0.1%)	13 (1.4%)
Patients reporting multiple symptoms *N* = 255/766 (28%)	190 (20.8%)	152 (16.6%)	140 (15.3%)	74 (8.1%)	20 (2.2%)	6 (0.7%)	44 (4.8%)
Total reporting symptoms *N* = 766/915 (84%)	45.6%	31.2%	19.6%	17.3%	3.6%	0.8%	6.2%

Perceptual difficulties inclusive of: depth perception difficulty, alexia, agraphia, photophobia, color perception difficulties.

### 3.7 Management

Treatment options were offered to all patients with visual impairment (92%: Table 6). Referral for new or updated refraction was most commonly provided (29.3%, *n* = 247) followed by prisms (12%, *n* = 101), occlusion (7.8%, *n* = 66), typoscopes (8.9%, *n* = 75), and low vision aids (3.8%, *n* = 32). Advice was offered to almost all patients (99%) and consisted of information about eye and head scanning training, reading strategies, appropriate lighting, visual field awareness, visual inattention awareness, and use of compensatory head posture.

**Table 6:**
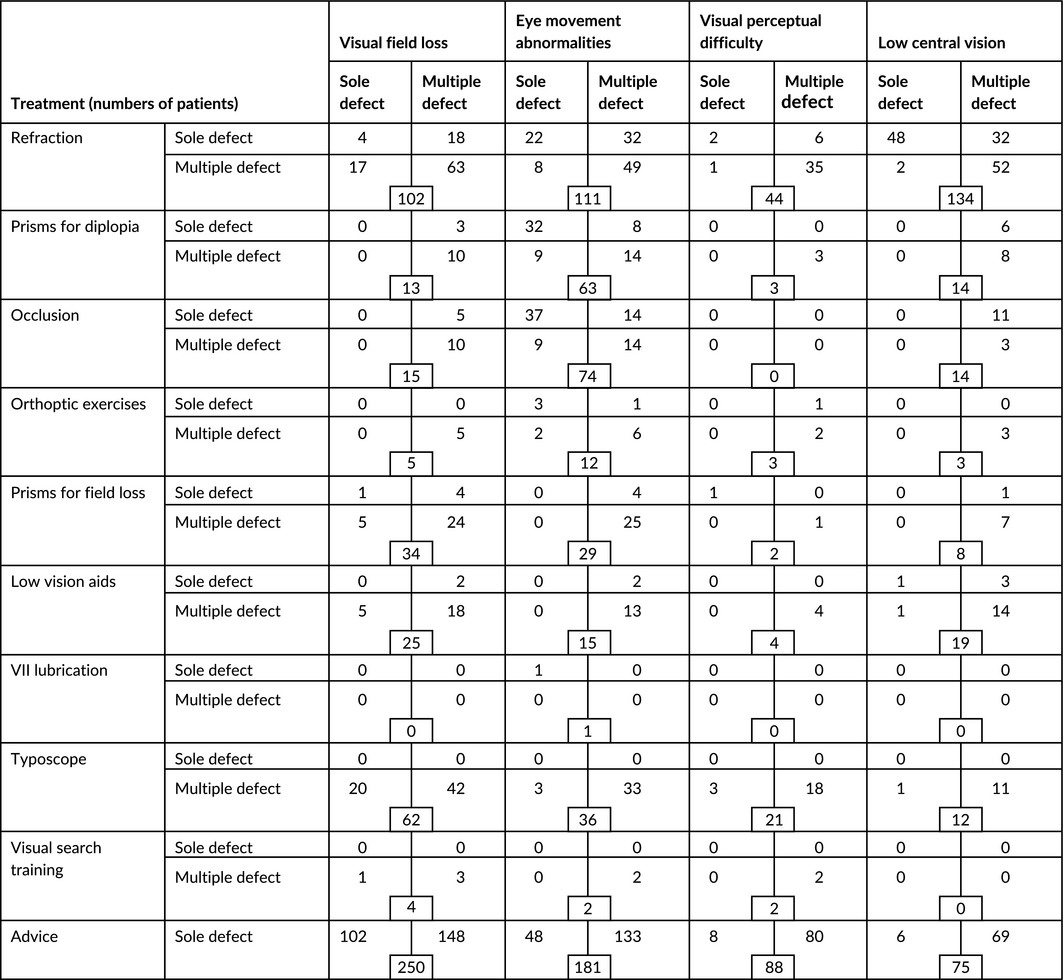
Treatment options across visual function categories

### 3.8 Impact to activities of daily living

**Figure 2:**
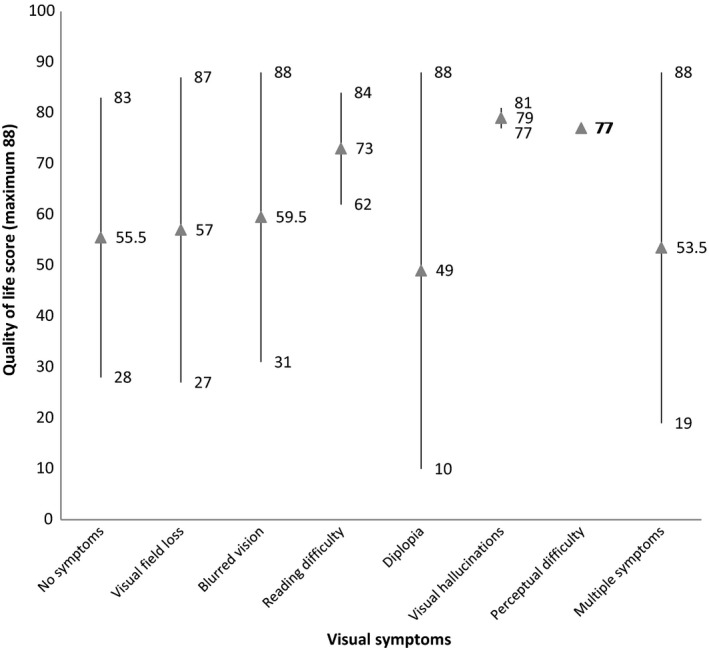
ADLDV quality of life scores

Activities of daily living dependent on vision were assessed for patients with and without visual symptoms (Figure 2). There was no significant difference between groups, *p* = .447 (Pearson *x* ^2^ test).

## 4 DISCUSSION

This study comprised a subpopulation of stroke survivors; those referred with suspected visual impairment. A high percentage of stroke survivors were subsequently confirmed as having visual impairment (92%). Types of visual impairment included visual field loss, ocular motility disorders, reduced central visual acuity, and visual perceptual disorders. Visual impairment most likely to be new and due to the stroke included visual field loss, ocular motility disorders, and visual perceptual disorders. Complete homonymous hemianopia was the most common form of visual field loss with partial hemianopia and quadrantanopia visual field defects also occurring frequently. Saccadic dysmetria was the most common form of ocular motility disorder with other frequently occurring disorders including cranial nerve palsy, gaze palsy, strabismus, reduced near point of convergence, and nystagmus. As expected, visual inattention was the most commonly occurring visual perceptual disorders but, in addition, patients were noted to report cortical impairment of color perception or depth, alexia, and visual agnosia. Fifty stroke survivors had normal visual assessment and were visually asymptomatic but, potentially, these may previously have been visually symptomatic but had recovery of their visual impairment by the time of undergoing visual assessment.

Reduced visual acuity of worse than 0.3 logMAR was noted in 24% of stroke survivors. Impaired central vision is often likely to precede a stroke and be due to coexistent ocular problems. For this cohort of stroke survivors, glasses were a common requirement for near and/or distance vision and coexistent or childhood ocular problems were noted in about one‐third of these patients. Similarly the finding of relative afferent pupillary defect and anisocoria were commonly related to ocular problems and less likely to be new onset related to stroke unlike pupil conditions such as Horner's syndrome and light‐near dissociation which were stroke‐related.

Diagnosis and confirmation of both new onset visual impairment and preexistent visual impairment are equally important; to ensure new onset visual impairment is accurately assessed and managed and to ensure that those with preexistent visual impairment continue with any previously ordered management program. Maximizing remaining visual function is essential to aid general rehabilitation.

Clearly, focused investigation is required to confirm the diagnosis of many of these disorders, particularly those with more subtle presentation features. Visual symptoms were reported overall by 84% of patients but of a wide variety as reported previously (Rowe, 2013). Notably, of 16% who were asymptomatic, many had confirmed visual impairment including substantial hemianopic visual field loss, limited eye movements, and reduced visual acuity. This latter group raises the question of how many stroke survivors with visual impairment may remain undetected where formal vision screening is not undertaken. This study only recruited those referred with suspected visual impairment and used a screening/referral form to facilitate this. However, as published previously, issues exist with such screening forms particularly where there is reliance on patient‐reported visual symptoms to aid identification of suspected visual impairment (Rowe, 2011). It remains unknown how many patients with visual impairment were undetected because of patient failure to report visual symptoms either because of communication, cognitive, or other failure to notify staff/carers of their symptoms and visual difficulties. Only specialist widespread visual screening of all stroke survivors will aid capture of such cases.

Given that poststroke visual impairment is estimated at 65% (Hepworth et al., 2015) and in view of the confirmed unmet needs reported by stroke survivors with visual impairment (Rowe et al., 2015) there is an urgent requirement to implement comprehensive, wide‐spread screening of stroke survivors to ensure identification of their visual issues. National guidance exists for provision of specialist services on stroke units for poststroke visual impairment with orthoptists being recommended as part of the core acute stroke unit team (British Irish Orthoptic Society, 2016; Intercollegiate Stroke Working Party, 2016); services that are proven to be feasible and acceptable for delivery on acute stroke units and neuro‐rehabilitation units and which are cost effective (Pollock, Hazelton, & Brady, 2011; Rowe et al., 2016). Recent research reports vision screening to be achievable at a median of 3 days post stroke with full visual examinations achieved at a median of 4 days post stroke (Rowe, Hepworth, Hanna, & Howard, 2016).

The impact of visual impairment is clear with considerable issues relating to driving, activities of daily living, mobilization, social engagement among others (Hepworth & Rowe, 2016). Given the high rate of visual symptoms and known impact, access to appropriate management options at the early acute poststroke stage is important. A wide variety of management options were provided in this study, many of which are evidence based as to their efficacy (Adler, 2002; Carruthers, Kennedy, & Bagaric, 1990; Firth & Whittle, 1994; Pollock et al., 2011, 2011a, 2011b, 2012; Thurtell & Leigh, 2010). It is important to note that, for advice on compensatory mechanisms, these are frequently of benefit to patients despite lacking an evidence base through case control or randomized trials. It is of further importance to recognize these management options are not ‘one size fits all’ but require targeting to the type of visual impairment and individual symptoms. Ensuring referral to specialist services will direct the stroke survivor to correct and appropriate treatment, whilst minimizing risks from inappropriate therapies provided by staff lacking the requisite training and/or knowledge.

## 5 CONCLUSIONS

The Vision In Stroke study is, to our knowledge, the first large‐scale observation study of poststroke visual impairment. In this population of stroke survivors referred with suspected visual impairment, prevalence of visual impairment was 92%. There are a wide range of visual disorders that occur following stroke and, frequently, give rise to visual symptoms. There are equally a wide variety of treatment options available for these individuals. We recommend that all stroke survivors require screening for visual impairment in the early days poststroke onset and warrant referral for specialist assessment and targeted treatment specific to the type of visual impairment.

## CONFLICT OF INTEREST

The authors have no disclosures or conflicts of interests.

## APPENDIX 1

### 1.1

VIS Writing Group: David Wright, Altnagelvin: Altnagelvin Hospitals HHS Trust; Darren Brand, Ayr: NHS Ayrshire and Arran; Tallat Maan, Durham: Durham and Darlington Hospitals NHS Foundation Trust; Sarah Peel, Jersey: St Helier General Hospital; Nicola Akerman, Nottingham: University Hospital NHS Trust; Caroline Dodridge, Oxford, Oxford Radcliffe Hospitals NHS Trust; Claire Howard, Salford: Salford Primary Care Trust; Tracey Shipman, Sheffield: Sheffield Teaching Hospitals NHS Foundation Trust; Una Sperring, Swindon: Swindon and Marlborough NHS Trust; Sonia MacDiarmid, Wigan: Wrightington, Wigan and Leigh NHS Trust; Cicely Freeman, Worcester: Worcestershire Acute Hospitals NHS Trust.
